# Cefazolin Versus Flucloxacillin for Methicillin-Susceptible *Staphylococcus aureus* Bone and Joint Infections: A Retrospective Single-Center Comparative Study of Effectiveness and Renal Safety of Intravenous Monotherapy

**DOI:** 10.3390/antibiotics15080798

**Published:** 2026-08-17

**Authors:** Felix Werneburg, Juliane Beschauner, Laura Isabell Werneburg, Alexander Zeh, Natalia Gutteck, Karl-Stefan Delank

**Affiliations:** 1 Department of Orthopedic and Trauma Surgery, Martin Luther University Halle-Wittenberg, Ernst-Grube-Straße 40, 06120 Halle (Saale), Germany; 2 Department of Prosthodontics and Geriatric Dentistry, Martin Luther University Halle-Wittenberg, Magdeburger Straße 16, 06112 Halle (Saale), Germany

**Keywords:** methicillin-susceptible *Staphylococcus aureus*, cefazolin, flucloxacillin, bone and joint infection, osteomyelitis, prosthetic joint infection, acute kidney injury, nephrotoxicity, antimicrobial stewardship

## Abstract

**Background/Objectives:** In methicillin-susceptible *Staphylococcus aureus* (MSSA) bacteremia, randomized evidence indicates comparable efficacy of cefazolin and antistaphylococcal penicillins with less nephrotoxicity; whether this extends to bone and joint infection (BJI) is unknown. We compared both agents in MSSA BJI. **Methods:** We retrospectively analyzed all adults at a single center with culture-confirmed, monomicrobial MSSA BJI treated with inpatient intravenous cefazolin or flucloxacillin monotherapy (January 2022–January 2025)—a selected population excluding rifampicin-based combination therapy, outpatient parenteral therapy, polymicrobial infection, and concurrent bacteremia/endocarditis. Endpoints, a priori exploratory, comprised effectiveness (mortality; clinical success) and renal safety (peri-treatment acute kidney injury [AKI]); the AKI comparison was additionally adjusted for confounders. **Results:** Among 110 patients (64 cefazolin, 46 flucloxacillin), baseline characteristics were comparable except for more frequent nephrotoxic co-medication with cefazolin (56.2% vs. 26.1%; *p* = 0.002). No statistically significant differences in effectiveness were detected: 30-day clinical success was 89.1% versus 80.4% (ARD 8.6 percentage points, 95% CI −4.7 to +23.3) and one-year clinical success 79.7% versus 65.2% (ARD 14.5, 95% CI −2.2 to +31.0); these imprecise estimates are compatible with effects ranging from no difference to a clinically relevant benefit of cefazolin. Peri-treatment AKI occurred in 19.0% versus 30.4% (ARD 11.4, 95% CI −4.7 to +27.7; adjusted odds ratio 2.27, 95% CI 0.87–5.91); the difference was confined to stage 1. **Conclusions:** In this selected inpatient monotherapy cohort, cefazolin was associated with numerically fewer AKI events, without statistically significant differences in any comparison. These exploratory findings support cefazolin as a rational targeted option for MSSA BJI, pending prospective confirmation.

## 1. Introduction

Methicillin-susceptible *Staphylococcus aureus* (MSSA) is one of the most common pathogens in bone and joint infection (BJI), including native and vertebral osteomyelitis, septic arthritis, fracture-related infection and prosthetic joint infection (PJI) [1,2]. The incidence of osteoarticular infection is rising as populations age and the use of orthopedic implants expands, and these infections impose a substantial clinical and economic burden through prolonged hospitalization, repeated surgery and extended courses of antibiotics [3,4]. For deep-seated staphylococcal infection, targeted therapy has traditionally relied on the antistaphylococcal penicillins (ASPs)—flucloxacillin, cloxacillin, oxacillin or nafcillin—or the first-generation cephalosporin cefazolin. Both are bactericidal β-lactams with reliable anti-MSSA activity, and for decades, the choice between them has rested more on institutional habit and regional availability than on high-quality comparative evidence [5].

The two agents differ in ways that matter for the prolonged, high-dose courses required in BJI. Cefazolin offers convenient every-eight-hour dosing that facilitates outpatient parenteral administration, a favorable tolerability profile, and an absence of the idiosyncratic hepatotoxicity that complicates flucloxacillin therapy—the latter now firmly linked to the HLA-B*57:01 genotype and quantified in population-based cohorts [6,7]. Its principal theoretical liability is the cefazolin inoculum effect (CzIE)—accelerated hydrolysis by certain type-A β-lactamases at the high bacterial densities characteristic of undrained or deep-seated infection—which has been proposed as a mechanism of microbiological failure [8]. Flucloxacillin, although not subject to the inoculum effect, requires four- to six-hourly dosing and has repeatedly been associated with acute kidney injury (AKI), particularly when co-administered with other nephrotoxins such as aminoglycosides [9,10]. Pharmacokinetically, cefazolin nonetheless achieves bone and synovial concentrations that reliably exceed staphylococcal minimum inhibitory concentrations, providing a rational basis for its use in osteoarticular infection [11,12].

In MSSA bacteremia, a consistent body of observational data and meta-analyses has favored cefazolin, reporting lower mortality and fewer treatment discontinuations for adverse events relative to ASPs [13,14]. This evidence culminated in the *Staphylococcus aureus* Network Adaptive Platform (SNAP) trial, which randomized more than 1200 patients with MSSA bacteremia and found cefazolin non-inferior to flucloxacillin or cloxacillin for 90-day mortality while causing significantly less AKI [15]. These findings, however, derive from bloodstream infection and cannot simply be assumed to transfer to osteoarticular disease.

Whether these conclusions extend to BJI is not self-evident. Osteoarticular infection differs from bacteremia in ways that could modify both effectiveness and safety: outcomes are dominated by the adequacy of surgical source control, by implant retention or removal, and by infection chronicity; therapy is given at high dose for weeks and followed by prolonged oral treatment; and the bacterial burden within necrotic bone, sequestra and implant-associated biofilm is high. Evidence from bacteremia therefore provides context for, rather than validation of, treatment decisions in BJI. Direct comparative data in BJI remain scarce and are largely confined to selected syndromes such as spinal epidural abscess [16,17]. We therefore conducted a retrospective comparative study to examine whether comparable effectiveness and a more favorable renal safety profile for cefazolin also apply in MSSA bone and joint infection, evaluating both clinical effectiveness and renal safety.

## 2. Results

### 2.1. Study Population and Baseline Characteristics

**Figure 1:**
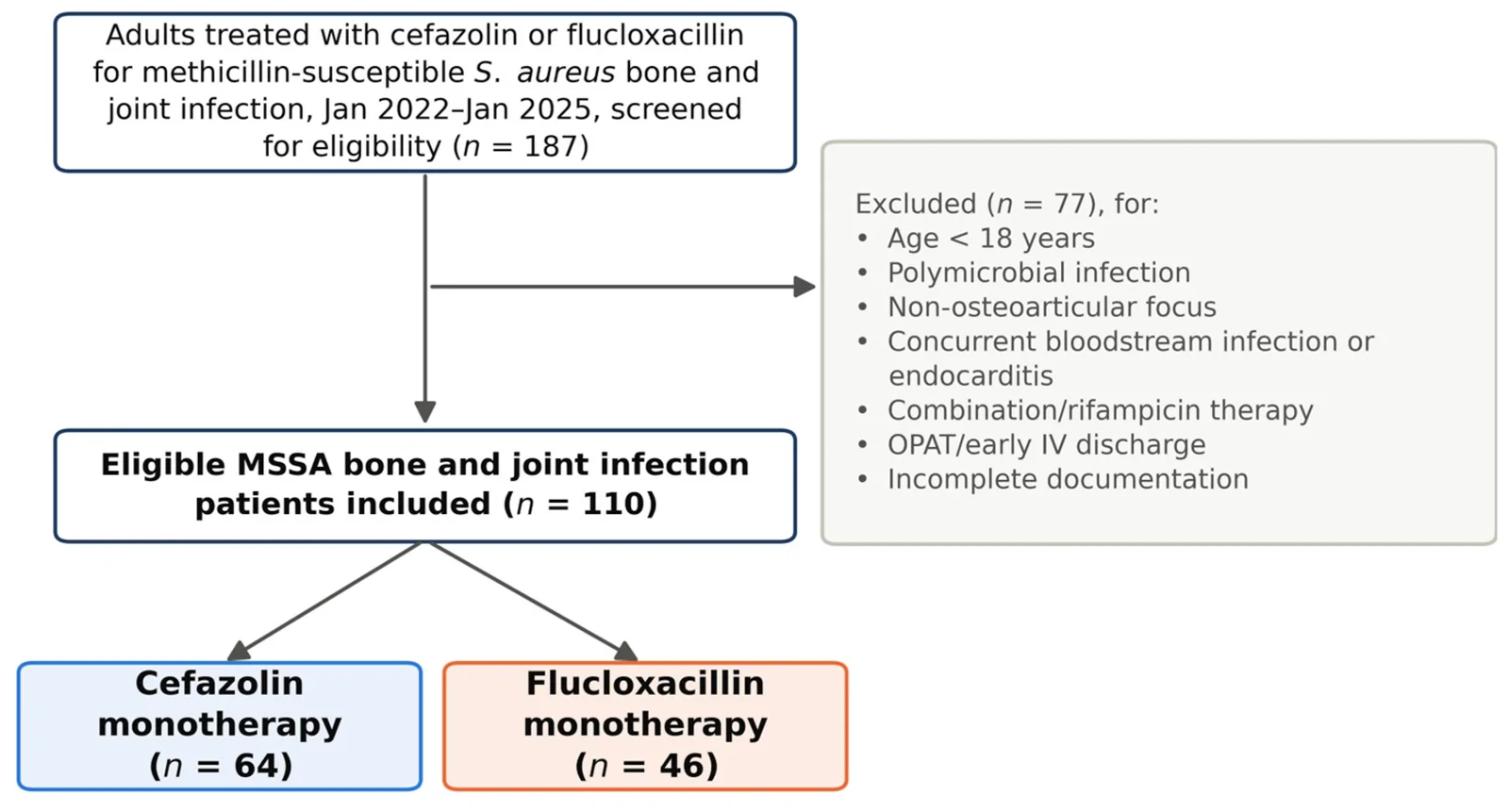
Study flow diagram. MSSA, methicillin-susceptible *Staphylococcus aureus*; OPAT, outpatient parenteral antimicrobial therapy; IV, intravenous.

Of 187 patients screened between January 2022 and January 2025, 110 adults with culture-confirmed, monomicrobial MSSA BJI met the eligibility criteria (77 were excluded): 64 treated with cefazolin monotherapy and 46 with flucloxacillin monotherapy (Figure 1). Baseline demographic, anthropometric and renal characteristics were largely comparable between groups (Table 1). Median age was 65.5 years (interquartile range [IQR] 57.8–75.0) in the cefazolin group and 66.5 years (IQR 60.0–77.0) in the flucloxacillin group (*p* = 0.67); the proportion of patients with an American Society of Anesthesiologists (ASA) physical status of III or IV was 60.9% versus 45.7% (*p* = 0.13). Prosthetic joint infection was the most frequent indication in both groups. Baseline estimated glomerular filtration rate (eGFR) and serum creatinine did not differ significantly.

The single notable imbalance concerned concomitant nephrotoxic medication, which was documented significantly more often in the cefazolin group than in the flucloxacillin group (36/64, 56.2% vs. 12/46, 26.1%; *p* = 0.002). Because this imbalance would be expected to bias against cefazolin with respect to the renal safety endpoint, it was addressed explicitly in the adjusted analysis (Section 2.3).

### 2.2. Effectiveness Outcomes

**Figure 2:**
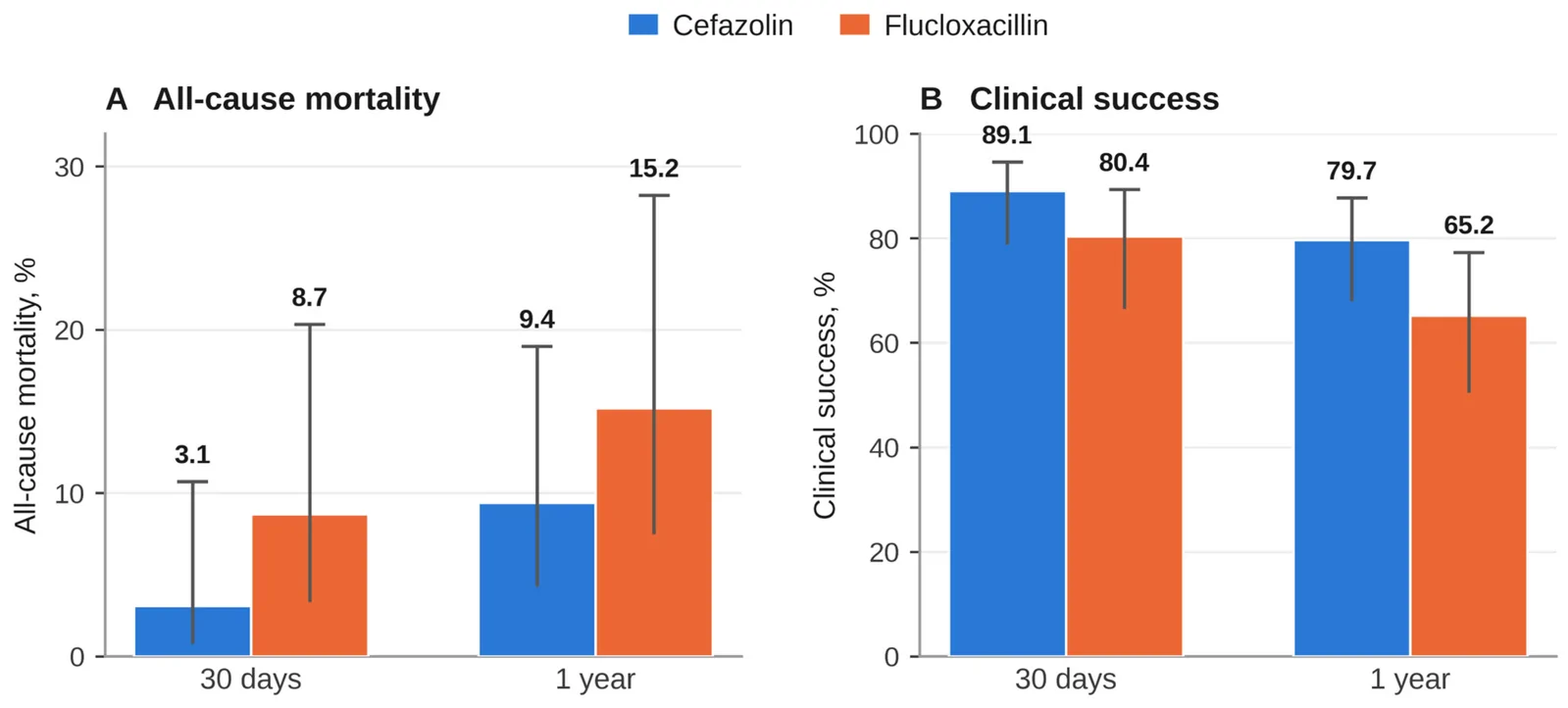
Clinical effectiveness at 30 days and 1 year by treatment group. (**A**) All-cause mortality, analyzed cumulatively: a patient who died within 30 days is also counted as deceased at one year. (**B**) Clinical success, defined as being alive and free of infection. Bars show percentages with Wilson 95% confidence intervals.

At 30 days, all-cause mortality was low in both arms and did not differ significantly: 2 of 64 patients (3.1%) in the cefazolin group versus 4 of 46 (8.7%) in the flucloxacillin group (odds ratio [OR] 2.65, 95% CI 0.54–13.04; *p* = 0.23) (Figure 2A, Table 2). With few deaths, this comparison is underpowered and is reported descriptively; the point estimate is directionally consistent with randomized data in bacteremia. Short-term clinical effectiveness, captured as freedom from infection at 30 days, was high: among survivors, 57 of 62 cefazolin-treated patients (91.9%) and 37 of 42 flucloxacillin-treated patients (88.1%) were infection-free (*p* = 0.52). Incorporating death as treatment failure, the composite of 30-day clinical success (survival free of infection) was met by 57 of 64 patients (89.1%) with cefazolin versus 37 of 46 (80.4%) with flucloxacillin (OR for clinical failure 1.94, 95% CI 0.69–5.51; *p* = 0.27) (Figure 2B). Length of hospital stay was similar between groups (median 17.5 vs. 16.0 days; *p* = 0.19).

At one year, the same pattern persisted. Cumulative all-cause mortality was 6 of 64 (9.4%) with cefazolin versus 7 of 46 (15.2%) with flucloxacillin (OR 1.71, 95% CI 0.55–5.26; *p* = 0.38) (Figure 2A). Among the survivors, 51 of 58 cefazolin-treated patients (87.9%) and 30 of 39 flucloxacillin-treated patients (76.9%) were free of infection at one year (*p* = 0.17). The composite of one-year clinical success (alive and infection-free) was met by 51 of 64 patients (79.7%) with cefazolin versus 30 of 46 (65.2%) with flucloxacillin (OR for clinical failure 2.06, 95% CI 0.88–4.82; *p* = 0.12) (Figure 2B). Expressed as absolute risk differences (flucloxacillin minus cefazolin), the between-group differences were +5.6 percentage points (95% CI −3.7 to +17.4) for 30-day mortality, +8.6 (−4.7 to +23.3) for 30-day clinical failure, +5.8 (−6.4 to +19.8) for one-year mortality and +14.5 percentage points (95% CI −2.2 to +31.0) for one-year clinical failure (Table 2). None of these comparisons reached statistical significance; the point estimates favored cefazolin at both time points, but the confidence intervals were wide and include effects ranging from no meaningful difference to a clinically relevant advantage.

The composition of clinical failure was as follows. At 30 days, failures comprised two deaths and five survivors with documented persistent or recurrent infection in the cefazolin group, versus four deaths and five such survivors in the flucloxacillin group. At one year, failures comprised six deaths, four survivors with documented persistent or recurrent infection, and three survivors classified conservatively as failures solely because freedom from infection was not positively documented, versus seven, four and five, respectively. A finer subdivision of non-fatal failures (microbiological relapse, unplanned reoperation, renewed antimicrobial therapy) was not retrievable from the source data; the impact of the conservative classification rule was examined in a sensitivity analysis (Section 2.5).

### 2.3. Renal Safety Outcomes

**Figure 3:**
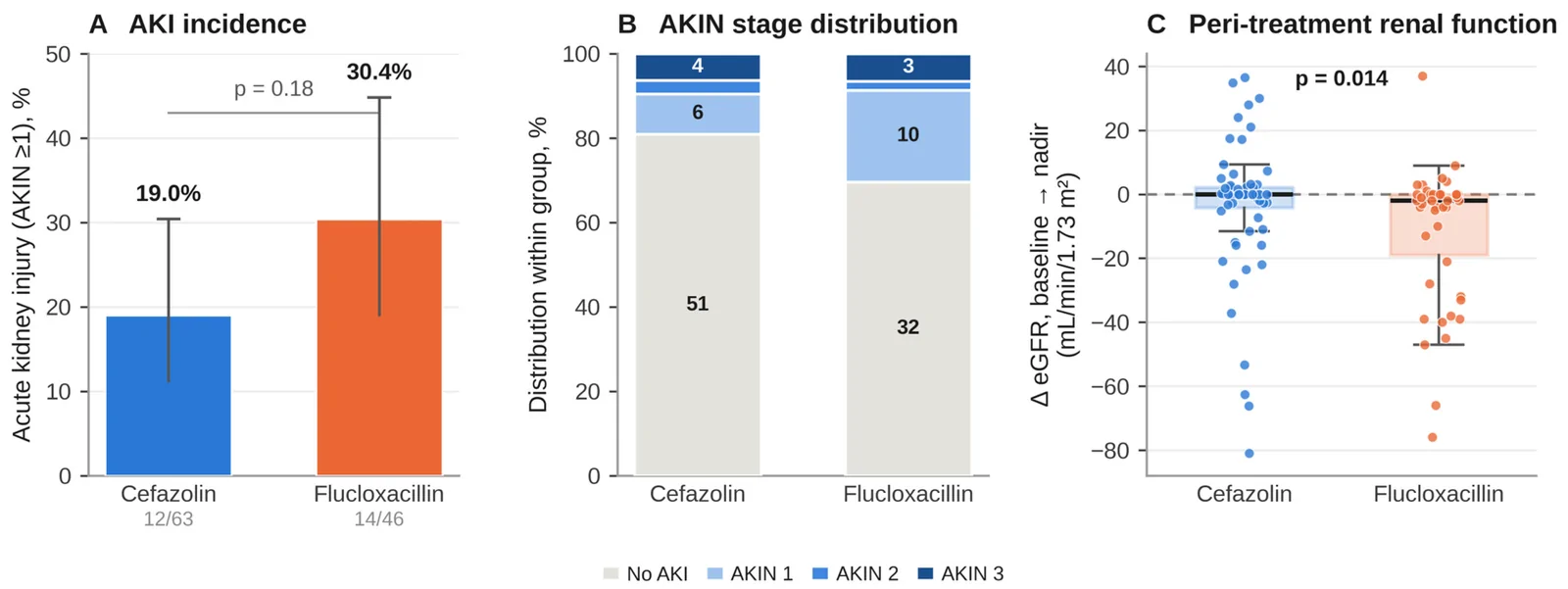
Renal safety by treatment group. (**A**) Incidence of acute kidney injury (AKI; stage ≥ 1) with Wilson 95% confidence intervals; *p* from Fisher’s exact test. (**B**) Distribution of AKI stages within each group (counts shown within bars); the between-group difference is driven mainly by stage 1 injury. (**C**) Peri-treatment change in estimated glomerular filtration rate (eGFR) from baseline to the on-treatment nadir (boxes, median and interquartile range; points, individual patients; dashed line, no change; *p* from the Mann–Whitney U test). Baseline eGFR was right-censored at 90 mL/min/1.73 m^2^ by the reporting laboratory. AKI staging followed a modified KDIGO/AKIN serum creatinine definition (Section 4.5).

During the inpatient stay on intravenous antibiotic therapy, peri-treatment acute kidney injury (AKI) occurred in 12 of 63 evaluable cefazolin patients (19.0%) and 14 of 46 flucloxacillin patients (30.4%) (OR 1.84, 95% CI 0.77–4.41; absolute risk difference +11.4 percentage points; *p* = 0.18) (Figure 3A). The distribution of AKI stages suggested that the excess in the flucloxacillin group was accounted for mainly by stage 1 (milder) injury, whereas moderate-to-severe AKI (stage ≥ 2) was similar between groups (9.5% vs. 8.7%) (Figure 3B).

A continuous measure of renal function pointed in the same direction: the peri-treatment decline in eGFR from baseline to nadir was greater in the flucloxacillin group (median change −2.0, mean −10.9 mL/min/1.73 m^2^) than in the cefazolin group (median 0.0, mean −3.7 mL/min/1.73 m^2^; *p* = 0.014) (Figure 3C). This eGFR comparison should, however, be read cautiously: baseline eGFR was right-censored at 90 mL/min/1.73 m^2^ by the reporting laboratory, and 22 of 64 cefazolin patients (34.4%) versus 19 of 46 flucloxacillin patients (41.3%) already lay at this ceiling at baseline, structurally predisposing the higher-baseline flucloxacillin group to larger apparent declines. In a sensitivity analysis restricted to the 69 patients with baseline eGFR below the ceiling (68 evaluable for the change in eGFR), the decline remained directionally greater with flucloxacillin (median −4.0 versus +0.2 mL/min/1.73 m^2^) but was no longer statistically significant (*p* = 0.078). The uncensored measure—the absolute rise in peak serum creatinine—was numerically but not significantly larger with flucloxacillin (median +6 vs. +3 µmol/L; *p* = 0.14).

### 2.4. Adjusted Analysis of AKI

**Figure 4:**
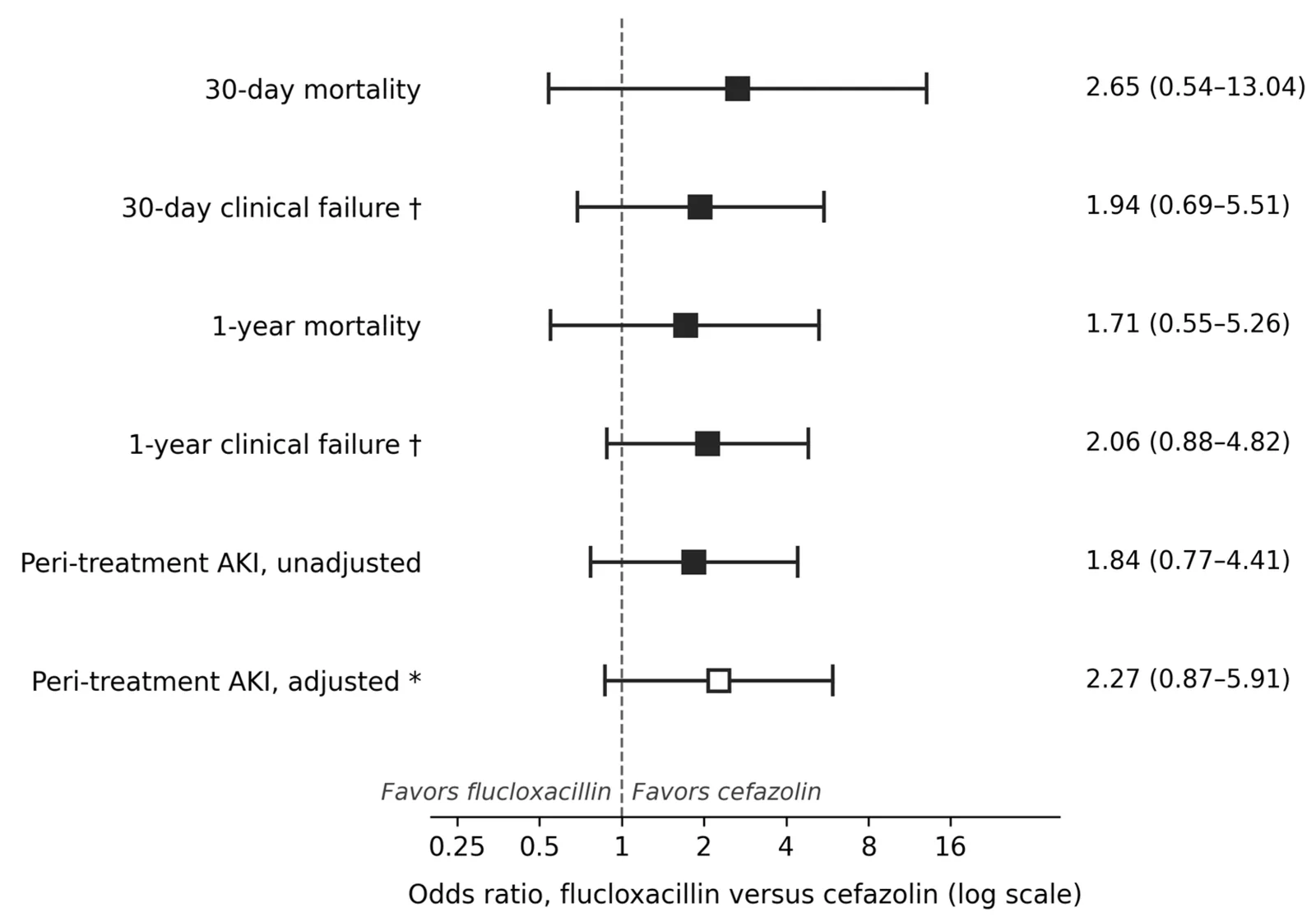
Odds ratios across outcomes, oriented as flucloxacillin versus cefazolin, such that values > 1 favor cefazolin. Error bars denote 95% confidence intervals. * Adjusted for nephrotoxic co-medication (parsimonious model; Section 4.6); exploratory. † Clinical failure = death or not infection-free. All endpoints were designated a priori as exploratory; no comparison reached statistical significance. AKI, acute kidney injury. Filled squares, unadjusted estimates; open square, adjusted estimate.

Because nephrotoxic co-medication—an established risk factor for AKI—was paradoxically more common in the cefazolin group, we fitted multivariable logistic-regression models to estimate the antibiotic-associated AKI risk independent of this confounding (Table 3). In the parsimonious model adjusting for nephrotoxic co-medication, flucloxacillin was associated with more than a doubling of the odds of AKI (aOR 2.27, 95% CI 0.87–5.91; *p* = 0.09). In the fully adjusted model additionally accounting for age and baseline eGFR, the point estimate increased (aOR 2.58, 95% CI 0.95–7.00; *p* = 0.06) but remained non-significant. Adjustment for the baseline imbalance therefore increased, rather than attenuated, the estimated association against flucloxacillin (Figure 4); given only 26 events, these adjusted estimates are exploratory (Section 4.6).

### 2.5. Sensitivity Analyses

To address the heterogeneity of infection syndromes, the principal endpoints were re-examined after stratification by implant status (implant-associated: prosthetic joint and fracture-related/implant-associated infection, *n* = 63; native: septic arthritis, native vertebral and long-bone osteomyelitis, *n* = 47) (Table S1). One-year clinical success favored cefazolin in both strata (implant-associated 82.5% vs. 69.6%; native 75.0% vs. 60.9%), with no evidence of effect modification (interaction *p* = 0.94); the Mantel–Haenszel odds ratio for one-year clinical failure pooled across strata (2.00, 95% CI 0.84–4.75) was virtually identical to the unadjusted estimate, indicating that the unequal syndrome distribution does not account for the effectiveness findings. For peri-treatment AKI, the between-group difference was concentrated in native infections (12.5% vs. 39.1%) and absent in implant-associated infections (23.1% vs. 21.7%); the treatment-by-stratum interaction was not statistically significant (*p* = 0.11), and the pooled Mantel–Haenszel estimate (OR 1.84, 95% CI 0.75–4.49) mirrored the unadjusted analysis. Given the small strata, these subgroup observations are exploratory. Finally, when the eight survivors without positive documentation of infection resolution were reclassified as infection-free (best case), one-year clinical success was 84.4% with cefazolin versus 76.1% with flucloxacillin (OR for clinical failure 1.68, 95% CI 0.66–4.29), indicating that the conservative classification rule did not generate the observed direction of effect.

## 3. Discussion

In this retrospective single-center cohort of 110 adults with culture-confirmed, monomicrobial MSSA bone and joint infection treated with intravenous monotherapy, no statistically significant differences between cefazolin and flucloxacillin were detected in effectiveness or in categorical peri-treatment renal safety. Point estimates for mortality, clinical success and AKI were directionally consistent in favoring cefazolin, but all interval estimates were wide; the findings therefore constitute an imprecise, hypothesis-generating signal rather than evidence of similarity or superiority. The categorical between-group difference in AKI was confined to stage 1 injury, whereas moderate-to-severe AKI (stage ≥ 2) was essentially identical between arms (9.5% vs. 8.7%). The one-year window provides a clinically meaningful follow-up horizon that captures early relapse, and the magnitude of the one-year difference in clinical success (14.5 percentage points), although not statistically significant, would be clinically relevant if confirmed. To our knowledge, this is among the first studies to examine this comparison specifically in osteoarticular infection.

Our findings are directionally consistent with the wider MSSA bacteremia literature. Meta-analyses of bacteremia cohorts have reported lower mortality and fewer adverse-event discontinuations with cefazolin than with ASPs [13,14], a large head-to-head cohort of the exact drug pairing reached the same conclusion [18], and a recent randomized platform trial reported non-inferior mortality with 33% lower adjusted odds of AKI for cefazolin [15]. The magnitude of nephrotoxicity we observed with flucloxacillin (30.4%) is consonant with dedicated studies: Crochette and colleagues reported AKI in 34.2% of patients receiving cloxacillin-based regimens [19], and flucloxacillin—especially combined with aminoglycosides—has been associated with AKI in 13% or more of arthroplasty patients [9,10]. The isoxazolyl penicillins are recognized causes of acute interstitial nephritis and acute tubular injury, providing a plausible mechanism for the signal we detected. These external data should nonetheless be read as context rather than validation: bacteremia and osteoarticular infection differ fundamentally in the dominance of surgical source control, implant-associated biofilm, infection chronicity and treatment duration, and concordance of direction across different clinical syndromes does not establish transferability of effect.

A particular strength of the present analysis is that the baseline imbalance ran counter to the observed effect. Nephrotoxic co-medication was substantially more common in the cefazolin group, yet cefazolin patients experienced numerically less renal injury; adjustment for this confounder therefore increased rather than attenuated the estimated association between flucloxacillin and AKI (primary parsimonious model: adjusted odds ratio 2.27, 95% CI 0.87–5.91; fully adjusted 2.58), although both models remained non-significant. The nominally significant continuous eGFR result must be interpreted with particular caution: baseline eGFR was right-censored at 90 mL/min/1.73 m^2^ and was higher (nearer the ceiling) in the flucloxacillin arm, so a change-from-baseline metric is structurally predisposed to show larger declines in that group; consistent with this, the uncensored measure—absolute creatinine rise—did not differ significantly (*p* = 0.14), and restriction to patients with baseline eGFR below the ceiling preserved the direction of the difference while attenuating it below statistical significance (*p* = 0.078). We therefore regard the eGFR-change finding as hypothesis-generating rather than as firm evidence of harm. That said, even mild, apparently reversible stage 1 AKI is associated with longer admissions, incident chronic kidney disease and increased downstream mortality, so a reproducible reduction would be a legitimate stewardship objective in a population already burdened by prolonged antibiotic exposure and repeated surgery.

The sensitivity analyses strengthen the internal consistency of these observations without altering their exploratory character. Stratification by implant status left the effectiveness estimates essentially unchanged, indicating that the unequal distribution of infection syndromes does not account for the observed direction, and the best-case reclassification of survivors without documented infection status likewise preserved it, arguing against differential outcome ascertainment as its principal explanation. For AKI, the between-group difference was concentrated in native infections and absent in implant-associated infections; given the small strata and a non-significant interaction, we regard this heterogeneity as exploratory and refrain from causal interpretation.

The chief theoretical argument against cefazolin in deep-seated infection—the cefazolin inoculum effect—did not manifest as a detectable clinical signal in our cohort. This observation must be interpreted cautiously: the CzIE was not tested microbiologically, and the study was not powered for microbiological failure or relapse, so the absence of a detectable clinical signal is not evidence that the mechanism was absent. Our observation nonetheless accords with a recent systematic review of 23 studies concluding that the CzIE did not predict higher mortality and does not currently justify routine laboratory testing [20]. It is also biologically consistent with the high bone and synovial concentrations that cefazolin attains in orthopedic patients [11,12]. The absence of any effectiveness signal against cefazolin—together with its practical advantages of eight-hourly dosing, better tolerability and suitability for outpatient parenteral therapy [5]—supports cefazolin as a rational targeted option for MSSA BJI, in line with the direction of the limited existing osteoarticular comparisons [16,17]. These considerations are directly relevant to the intravenous-to-oral switch and outpatient strategies that now shape BJI management [21], and complement rather than replace the syndrome-specific recommendations of current guidelines [22,23].

Several limitations must temper these conclusions. First, the study is retrospective, single-center and modest in size; no a priori sample-size calculation was performed and all eligible patients treated between January 2022 and January 2025 were included, so the cohort is a convenience sample that is underpowered for the mortality and categorical AKI comparisons and hypothesis-generating rather than confirmatory. Residual confounding cannot be excluded: baseline eGFR was numerically lower in the cefazolin arm (73.5 vs. 87.5 mL/min/1.73 m^2^; *p* = 0.16), and comorbidities strongly linked to both AKI and mortality—diabetes, pre-existing chronic kidney disease, sepsis severity and hemodynamic status—were not captured, nor were antibiotic treatment duration, the oral agents used after discharge, or the cause of death, so that only all-cause mortality could be assessed. The distribution of infection syndromes also differed between arms (for example, native vertebral osteomyelitis in 12.5% of cefazolin versus 24.0% of flucloxacillin patients), and the effectiveness endpoints—unlike the AKI analysis—were not adjusted for baseline imbalances beyond the stratified sensitivity analysis by implant status (Section 2.5).

Second, the attribution of renal events deserves particular emphasis. AKI was ascertained relative to the pre-operative baseline, whereas definitive cefazolin or flucloxacillin therapy commenced only after culture-based de-escalation from empirical ampicillin/sulbactam with or without vancomycin; renal events occurring early in the treatment episode may therefore reflect surgery, sepsis, hemodynamic factors, contrast exposure or the preceding empirical regimen rather than the definitive β-lactam. The source data, extracted at fixed peri-treatment time points without time-resolution relative to the treatment switch, do not permit re-anchoring the baseline to the start of definitive therapy; we have accordingly framed the endpoint as peri-treatment AKI without causal attribution to the study antibiotic. Two considerations mitigate, without eliminating, this concern: both arms passed through the same standardized surgical and empirical pathway, rendering such misattribution plausibly non-differential; and systemic vancomycin exposure during the empirical phase was captured within the nephrotoxic co-medication variable adjusted for in the regression models—a variable more prevalent in the cefazolin arm, so that misattributed vancomycin-associated injury would be expected to bias against cefazolin, the opposite of the observed direction. Time from initiation of definitive therapy to AKI, the duration of intravenous therapy, cumulative antibiotic doses and the individual nephrotoxic agents were not retrievable from the source data. In addition, AKI was ascertained from serial serum creatinine without urine-output data; because the timing of sampling was not standardized to the strict 48 h window, this represents a modified KDIGO/AKIN serum creatinine definition rather than formal AKIN staging, and unstandardized timing may misclassify AKI in either direction. One cefazolin patient had no post-operative creatinine and was non-evaluable for AKI (evaluable *n* = 63), and six further patients had only a single post-operative measurement; missing later values would, if anything, bias toward under-detection of AKI in the cefazolin arm. Creatinine was captured on a uniform peri-treatment schedule in both groups, arguing against differential monitoring between arms, although the limited number of measurements per patient may under-detect AKI overall. As noted, baseline eGFR was right-censored at 90 mL/min/1.73 m^2^, which biases the eGFR-change analysis against the higher-baseline flucloxacillin arm; nephrotoxic co-medication was captured only as a binary variable without dose, duration or agent-level resolution. Third, the requirement for intravenous monotherapy defines a deliberately selected cohort: patients who would ordinarily receive rifampicin combination therapy were excluded by design. In prosthetic joint infection, this meant excluding acute infections managed by debridement with implant retention and staged prosthesis re-implantations—settings in which rifampicin is routinely combined with the β-lactam—so that only patients treated with cefazolin or flucloxacillin monotherapy (for example, after implant removal, or where rifampicin was contraindicated) were included. This strengthens internal validity for the monotherapy question but limits generalizability to combination therapy settings. Finally, freedom from infection was a retrospectively abstracted, unblinded clinical judgment subject to ascertainment bias; only patients whose one-year vital status was documented were included, with undocumented freedom from infection among survivors counted conservatively as treatment failure. While the one-year window captures earlier relapse than the 30-day assessment, formally adjudicated microbiological cure, reoperation for infection and patient-reported functional outcomes—arguably the most relevant effectiveness endpoints in BJI—were not available, and later relapses beyond one year were not assessed.

These limitations define the agenda for confirmatory work. Adequately powered, prospective and ideally randomized studies designed for osteoarticular infection—incorporating treatment failure, relapse and patient-reported functional endpoints, standardized AKI ascertainment and, where feasible, characterization of the cefazolin inoculum effect—are needed to establish whether cefazolin should become the default targeted therapy for MSSA BJI.

## 4. Materials and Methods

### 4.1. Study Design and Setting

We performed a retrospective observational cohort study at the Department of Orthopedic and Trauma Surgery, Martin Luther University Halle-Wittenberg (a tertiary-care university center in Halle (Saale), Germany). All adult patients treated with intravenous cefazolin or flucloxacillin for MSSA bone and joint infection between January 2022 and January 2025 were screened for eligibility (187 patients screened, 77 excluded, 110 included; Figure 1). The study is reported in accordance with the Strengthening the Reporting of Observational Studies in Epidemiology (STROBE) guidelines [24]; the completed STROBE checklist is provided as Supplementary Material.

### 4.2. Participants

Eligible patients were aged 18 years or older with a culture-confirmed, monomicrobial MSSA bone or joint infection (native or vertebral osteomyelitis, septic arthritis, fracture-related/implant-associated infection, or prosthetic joint infection) who received intravenous monotherapy with either cefazolin or flucloxacillin and for whom the one-year vital status (survival or death) was documented. Patients were excluded if they had a polymicrobial infection; were treated for a concurrent non-osteoarticular focus (e.g., pneumonia or urinary-tract infection); had a concurrent *S. aureus* bloodstream infection or infective endocarditis; received combination antimicrobial therapy, in particular rifampicin-containing regimens used at prosthesis re-implantation; were discharged early on outpatient parenteral antimicrobial therapy (OPAT) via a peripherally inserted central catheter while still receiving intravenous antibiotics; or had incomplete clinical or laboratory documentation. The selection process is summarized in Figure 1.

MSSA was identified from intraoperative deep-tissue specimens and, where implants were removed, implant sonication fluid obtained at surgical debridement. Specimens were cultured on standard aerobic and anaerobic solid and enrichment media in the institutional microbiology laboratory, with an extended incubation of up to 14 days in keeping with recommendations for bone, joint and implant-associated infection. Species identification and antimicrobial susceptibility testing were performed according to current international standards, and susceptibility was interpreted according to the EUCAST clinical breakpoints in force at the time of testing (2022–2025). A direct microbiological pathogen detection that guided targeted antibiotic therapy was available for every included patient; monomicrobial infection was defined as growth of MSSA as the sole pathogen from the relevant deep specimens. The cefazolin inoculum effect was not routinely tested.

### 4.3. Treatment Protocol

After surgical debridement, patients received empirical calculated antibiotic therapy in line with current recommendations and the institutional standard operating procedure, comprising ampicillin/sulbactam with vancomycin added in patients with risk factors for methicillin-resistant *S. aureus* [25,26]. Once microbiological cultures identified monomicrobial MSSA, therapy was de-escalated to intravenous monotherapy with either cefazolin (typically 2 g every 8 h) or flucloxacillin (typically 2 g every 4–6 h), with doses adjusted for renal function. The choice between the two agents was made by the treating orthopedic and infectious-disease team on a case-by-case basis rather than by a protocolized allocation rule. Intravenous therapy was continued throughout the inpatient stay; at discharge, all patients were switched to an oral antimicrobial agent. Patients continuing intravenous therapy in the outpatient setting (OPAT) were not included, ensuring a homogeneous inpatient intravenous exposure.

### 4.4. Data Collection and Variables

Data were extracted from the electronic medical record. Collected variables comprised age, sex, body-mass index, ASA physical status, infection type, and the presence of nephrotoxic co-medication. Renal function was recorded at three time points—before surgery (baseline) and at two post-operative/peri-treatment measurements—as both serum creatinine (µmol/L) and eGFR (mL/min/1.73 m^2^). eGFR was estimated with the 2021 CKD-EPI creatinine equation (race-free) and reported by the central laboratory, right-censored at 90 mL/min/1.73 m^2^. Nephrotoxic co-medication was defined a priori, informed by a published nephrotoxin list [27], as the concomitant administration during antibiotic treatment of any of: non-steroidal anti-inflammatory drugs, aminoglycosides, angiotensin-converting-enzyme inhibitors or angiotensin-receptor blockers, or loop diuretics.

### 4.5. Outcomes and Definitions

The outcomes, all designated a priori as exploratory and hypothesis-generating, spanned two domains and were assessed at 30 days and at one year. Effectiveness comprised all-cause mortality and freedom from infection. Mortality was analyzed cumulatively: a patient who died within 30 days was also counted as deceased at one year. Freedom from infection was assessed among the patients alive at each time point, and a patient not classified as infection-free at 30 days was likewise counted as not infection-free at one year. In keeping with consensus outcome frameworks for prosthetic joint and fracture-related infection [22,26], freedom from infection was defined as the resolution and continued absence of persistent or recurrent infection—no ongoing clinical, biochemical or microbiological signs of infection—not requiring unplanned antimicrobial re-treatment or unplanned surgical re-intervention for infection. Crucially, planned staged procedures forming part of the intended treatment pathway (for example, debridement with spacer interposition followed by a scheduled joint reconstruction or arthroplasty in staged management) were not classified as treatment failures; conversely, an unplanned reoperation for infection, an infection-related death, or a microbiologically confirmed relapse constituted treatment failure. The composite endpoint of clinical success was defined as being alive and free of infection at the respective time point; deceased patients were counted as treatment failures. Only patients whose one-year vital status could be established were included; among survivors, freedom from infection required positive documentation of infection resolution, and survivors without such documentation were counted as not infection-free, so that all 110 patients were evaluable at both time points. Renal safety was captured by peri-treatment acute kidney injury (AKI), defined using a modified serum creatinine definition based on the Kidney Disease: Improving Global Outcomes (KDIGO)/Acute Kidney Injury Network (AKIN) criteria, with the pre-operative value as baseline and no urine-output data: stage 1, an increase of ≥26.5 µmol/L or to 1.5–1.9 times baseline; stage 2, 2.0–2.9 times baseline; and stage 3, ≥3.0 times baseline or an absolute value ≥ 353.6 µmol/L [28,29]. The peak on-treatment creatinine was compared with the pre-operative baseline; because routine sampling was not standardized to the strict 48 h window, staging reflects the peri-treatment interval rather than formal AKIN timing. Because the pre-operative value served as baseline and definitive cefazolin or flucloxacillin therapy commenced only after culture-based de-escalation from the empirical regimen, AKI is reported as a peri-treatment outcome of the overall treatment episode and is not causally attributed to the definitive study antibiotic; the implications of this design are addressed in the Discussion. Further secondary outcomes were length of hospital stay (capped at the 30-day observation window for the six patients who died within 30 days), AKI stage distribution, moderate-to-severe AKI (stage ≥ 2), and the change in eGFR from baseline to the on-treatment nadir.

### 4.6. Statistical Analysis

No a priori sample-size calculation was performed; all eligible patients over the study period were included. Continuous variables are summarized as median (IQR) and compared with the Mann–Whitney U test; categorical variables are summarized as counts (percentages) and compared with Fisher’s exact test. Ninety-five-percent confidence intervals for proportions were computed by the Wilson score method, and unadjusted odds ratios for 2 × 2 comparisons using the Haldane–Anscombe continuity correction with Woolf confidence intervals, each derived from the corresponding standard closed-form expressions. Between-group absolute risk differences for categorical endpoints are reported in percentage points with 95% confidence intervals by the Newcombe hybrid score method. To estimate the antibiotic-associated risk of AKI independent of the baseline imbalance in nephrotoxic co-medication, multivariable logistic-regression models were fitted with AKI as the dependent variable: a parsimonious model (antibiotic and nephrotoxic co-medication) and a fully adjusted model additionally including age and baseline eGFR. With 26 AKI events, the fully adjusted four-covariate model provides approximately 6.5 events per variable—below the conventional threshold of ten—so the parsimonious model was regarded as primary and the fully adjusted estimates as exploratory. Two-sided *p*-values < 0.05 were considered statistically significant, without adjustment for multiplicity given the hypothesis-generating intent; the single nominally significant result should be interpreted in that light. Sensitivity analyses added during revision comprised (i) stratification of the principal endpoints by implant status (implant-associated versus native infection), with Mantel–Haenszel pooled odds ratios and a multiplicative treatment-by-stratum interaction term in a logistic model; (ii) restriction of the eGFR-change analysis to patients with baseline eGFR below the 90 mL/min/1.73 m^2^ reporting ceiling; and (iii) a best-case reclassification in which survivors without positive documentation of infection resolution were counted as infection-free. Descriptive statistics, the Mann–Whitney U and Fisher’s exact tests, and the logistic-regression models were computed in IBM SPSS Statistics version 29 (IBM Corp., Armonk, NY, USA); these supplementary analyses were performed in Python 3.12 (Python Software Foundation, Wilmington, DE, USA; SciPy 1.17, statsmodels 0.14).

### 4.7. Ethics

The study was conducted in accordance with the Declaration of Helsinki. In accordance with the regulations of the Ethics Committee of the Medical Faculty of Martin Luther University Halle-Wittenberg and the applicable state legislation, formal ethical review and approval were not required for this retrospective analysis of fully anonymized routine clinical data; the requirement for individual informed consent was accordingly waived.

## 5. Conclusions

In this retrospective single-center cohort of adults with MSSA bone and joint infection treated with intravenous cefazolin or flucloxacillin monotherapy—a selected inpatient population excluding rifampicin-based combination regimens and outpatient parenteral therapy—no statistically significant differences in effectiveness or in categorical peri-treatment acute kidney injury were detected. Cefazolin was associated with numerically fewer AKI events, a signal that persisted after adjustment for the greater burden of nephrotoxic co-medication in that group and remained directionally consistent across sensitivity analyses, although all estimates were imprecise and potentially affected by treatment-selection bias, syndrome heterogeneity, preceding empirical therapy and unmeasured source control factors. These exploratory findings support cefazolin as a rational targeted option for MSSA BJI, but do not establish it as the preferred first-line choice. Adequately powered prospective studies incorporating treatment failure, relapse and functional endpoints, standardized renal safety ascertainment anchored to the start of definitive therapy and, where feasible, characterization of the cefazolin inoculum effect are required.

## Figures and Tables

**Table 1:** Baseline characteristics of the study population. Continuous variables are shown as median (interquartile range); categorical variables as *n* (%). *p*-values from the Mann–Whitney U test (continuous) or Fisher’s exact test (categorical). eGFR, estimated glomerular filtration rate; ASA, American Society of Anesthesiologists physical status.

Characteristic	Cefazolin (*n* = 64)	Flucloxacillin (*n* = 46)	*p*
Age, years	65.5 (57.8–75.0)	66.5 (60.0–77.0)	0.67
Male sex	40 (62.5)	22 (47.8)	0.17
Body-mass index, kg/m^2^	27.6 (25.8–31.0)	27.0 (24.7–28.8)	0.30
ASA class III–IV	39 (60.9)	21 (45.7)	0.13
Baseline eGFR, mL/min/1.73 m^2^	73.5 (51.8–90.0)	87.5 (69.5–90.0)	0.16
Baseline serum creatinine, µmol/L	79.5 (65.8–103.8)	78.0 (71.0–94.8)	0.64
Nephrotoxic co-medication	36 (56.2)	12 (26.1)	0.002
Infection type			
Prosthetic joint infection	29 (45.3)	18 (39.1)	—
Septic (native joint) arthritis	11 (17.2)	6 (13.0)	—
Fracture-related/implant-associated	11 (17.2)	5 (10.9)	—
Native vertebral osteomyelitis	8 (12.5)	11 (24.0)	—
Long-bone osteomyelitis	5 (7.8)	6 (13.0)	—

**Table 2:** Effectiveness and renal safety outcomes. Odds ratios (ORs) are expressed for flucloxacillin versus cefazolin; values > 1 favor cefazolin. ¶ Absolute risk differences (ARDs) are expressed in percentage points as flucloxacillin minus cefazolin, so that positive values indicate more events with flucloxacillin; 95% confidence intervals by the Newcombe hybrid score method; for clinical success, the ARD refers to clinical failure. ‡ Clinical success was defined as being alive and infection-free at the respective time point; deceased patients were counted as treatment failures. § The odds ratio is shown for clinical failure (death or persistent/recurrent infection), for which values > 1 favor cefazolin. Survivors not documented as infection-free at one year were counted as not infection-free. AKI, acute kidney injury; eGFR, estimated glomerular filtration rate; IQR, interquartile range.

Outcome	Cefazolin	Flucloxacillin	ARD, pp (95% CI) ¶	Effect (95% CI)	*p*
Effectiveness—30 days					
All-cause mortality	2 (3.1%)	4 (8.7%)	+5.6 (−3.7 to +17.4)	OR 2.65 (0.54–13.04)	0.23
Infection-free (survivors)	57/62 (91.9%)	37/42 (88.1%)	—	—	0.52
Clinical success (composite) ‡	57 (89.1%)	37 (80.4%)	+8.6 (−4.7 to +23.3)	OR 1.94 (0.69–5.51) §	0.27
Effectiveness—1 year					
All-cause mortality (cumulative)	6 (9.4%)	7 (15.2%)	+5.8 (−6.4 to +19.8)	OR 1.71 (0.55–5.26)	0.38
Infection-free (survivors)	51/58 (87.9%)	30/39 (76.9%)	—	—	0.17
Clinical success (composite) ‡	51 (79.7%)	30 (65.2%)	+14.5 (−2.2 to +31.0)	OR 2.06 (0.88–4.82) §	0.12
Length of hospital stay, days (median, IQR)	17.5 (15.0–30.0)	16.0 (14.0–25.8)	—	—	0.19
Renal safety					
Peri-treatment AKI (stage ≥ 1)	12/63 (19.0%)	14/46 (30.4%)	+11.4 (−4.7 to +27.7)	OR 1.84 (0.77–4.41)	0.18
AKI (stage ≥ 2)	6 (9.5%)	4 (8.7%)	−0.8 (−11.9 to +11.9)	—	1.00
Change in eGFR to nadir (median)	0.0	−2.0	—	—	0.014
Peak creatinine rise, µmol/L (median)	+3	+6	—	—	0.14

**Table 3:** Multivariable logistic-regression models for peri-treatment acute kidney injury (*n* = 109 patients; 26 events). Adjusted odds ratios (aORs) are shown for each covariate; flucloxacillin is compared with cefazolin (reference). Age and eGFR are modeled per 10-unit increment. eGFR, estimated glomerular filtration rate.

Variable	aOR	95% CI	*p*
Model 1—parsimonious			
Flucloxacillin (vs. cefazolin)	2.27	0.87–5.91	0.09
Nephrotoxic co-medication	1.87	0.72–4.88	0.20
Model 2—fully adjusted			
Flucloxacillin (vs. cefazolin)	2.58	0.95–7.00	0.06
Nephrotoxic co-medication	1.71	0.63–4.62	0.29
Age, per 10 years	0.91	0.65–1.27	0.58
Baseline eGFR, per 10 mL/min/1.73 m^2^	0.84	0.67–1.05	0.13

## Data Availability

The anonymized data presented in this study are available on request from the corresponding author. The data are not publicly available owing to privacy restrictions.

## Supplementary Materials

The following supporting information can be downloaded at: https://www.mdpi.com/article/10.3390/antibiotics15080798/s1. Table S1: Sensitivity analysis of principal endpoints stratified by implant status; STROBE checklist for cohort studies.

## References

[1] Tong S.Y.C., Davis J.S., Eichenberger E., Holland T.L., Fowler V.G. (2015). *Staphylococcus aureus* infections: Epidemiology, pathophysiology, clinical manifestations, and management. Clin. Microbiol. Rev..

[2] Zimmerli W., Trampuz A., Ochsner P.E. (2004). Prosthetic-joint infections. N. Engl. J. Med..

[3] Kremers H.M., Nwojo M.E., Ransom J.E., Wood-Wentz C.M., Melton L.J., Huddleston P.M. (2015). Trends in the epidemiology of osteomyelitis: A population-based study, 1969 to 2009. J. Bone Jt. Surg. Am..

[4] Grammatico-Guillon L., Baron S., Gettner S., Lecuyer A.-I., Gaborit C., Rosset P., Rusch E., Bernard L. (2012). Bone and joint infections in hospitalized patients in France, 2008: Clinical and economic outcomes. J. Hosp. Infect..

[5] Loubet P., Burdet C., Vindrios W., Grall N., Wolff M., Yazdanpanah Y., Andremont A., Duval X., Lescure F.-X. (2018). Cefazolin versus anti-staphylococcal penicillins for treatment of methicillin-susceptible *Staphylococcus aureus* bacteraemia: A narrative review. Clin. Microbiol. Infect..

[6] Daly A.K., Donaldson P.T., Bhatnagar P., Shen Y., Pe’er I., Floratos A., Daly M.J., Goldstein D.B., John S., Nelson M.R. (2009). HLA-B*5701 genotype is a major determinant of drug-induced liver injury due to flucloxacillin. Nat. Genet..

[7] Wing K., Bhaskaran K., Pealing L., Root A., Smeeth L., van Staa T.P., Klungel O.H., Reynolds R.F., Douglas I. (2017). Quantification of the risk of liver injury associated with flucloxacillin: A UK population-based cohort study. J. Antimicrob. Chemother..

[8] Nannini E.C., Stryjewski M.E., Singh K.V., Bourgogne A., Rude T.H., Corey G.R., Fowler V.G., Murray B.E. (2009). Inoculum effect with cefazolin among clinical isolates of methicillin-susceptible *Staphylococcus aureus*: Frequency and possible cause of cefazolin treatment failure. Antimicrob. Agents Chemother..

[9] Graham J., Borthwick E., Hill C., Blaney J., Gallagher N., Armstrong L., Beverland D. (2020). Acute kidney injury following prophylactic flucloxacillin and gentamicin in primary hip and knee arthroplasty. Clin. Kidney J..

[10] Challagundla S.R., Knox D., Hawkins A., Hamilton D., Flynn R.W.V., Robertson S., Isles C. (2013). Renal impairment after high-dose flucloxacillin and single-dose gentamicin prophylaxis in patients undergoing elective hip and knee replacement. Nephrol. Dial. Transplant..

[11] Zeller V., Durand F., Kitzis M.-D., Lhotellier L., Ziza J.-M., Mamoudy P., Desplaces N. (2009). Continuous cefazolin infusion to treat bone and joint infections: Clinical efficacy, feasibility, safety, and serum and bone concentrations. Antimicrob. Agents Chemother..

[12] Cunha B.A., Gossling H.R., Pasternak H.S., Nightingale C.H., Quintiliani R. (1977). The penetration characteristics of cefazolin, cephalothin, and cephradine into bone in patients undergoing total hip replacement. J. Bone Jt. Surg. Am..

[13] Bidell M.R., Patel N., O’Donnell J.N. (2018). Optimal treatment of MSSA bacteraemias: A meta-analysis of cefazolin versus antistaphylococcal penicillins. J. Antimicrob. Chemother..

[14] Allen J.M., Bakare L., Casapao A.M., Klinker K., Childs-Kean L.M., Pomputius A.F. (2019). Cefazolin versus anti-staphylococcal penicillins for the treatment of patients with methicillin-susceptible *Staphylococcus aureus* infection: A meta-analysis with trial sequential analysis. Infect. Dis. Ther..

[15] Lee T.C., Barina L.A., Walls G., Goodman A.L., Yahav D., Cheng M.P., Bonten M., Bowen A.C., Boyles T., Daneman N. (2026). Cefazolin for methicillin-susceptible *Staphylococcus aureus* bacteremia. N. Engl. J. Med..

[16] Corsini Campioli C., Go J.R., Abu Saleh O., Challener D., Yetmar Z., Osmon D.R. (2021). Antistaphylococcal penicillin vs cefazolin for the treatment of methicillin-susceptible *Staphylococcus aureus* spinal epidural abscesses. Open Forum Infect. Dis..

[17] Bai A.D., Findlater A., Irfan N., Singhal N., Loeb M. (2021). Cefazolin versus cloxacillin as definitive antibiotic therapy for methicillin-susceptible *Staphylococcus aureus* spinal epidural abscess: A retrospective cohort study. Int. J. Antimicrob. Agents.

[18] Davis J.S., Turnidge J., Tong S.Y.C. (2018). A large retrospective cohort study of cefazolin compared with flucloxacillin for methicillin-susceptible *Staphylococcus aureus* bacteraemia. Int. J. Antimicrob. Agents.

[19] Crochette R., Ravaiau C., Perez L., Coindre J.P., Piccoli G.B., Blanchi S. (2021). Incidence and risk factors for acute kidney injury during the treatment of methicillin-sensitive *Staphylococcus aureus* infections with cloxacillin-based antibiotic regimens: A French retrospective study. J. Clin. Med..

[20] Lo C.K.-F., Sritharan A., Zhang J., Li N., Zhang C., Wang F., Loeb M., Bai A.D. (2024). Clinical significance of cefazolin inoculum effect in serious MSSA infections: A systematic review. JAC-Antimicrob. Resist..

[21] Li H.K., Rombach I., Zambellas R., Walker S., McNally M.A., Atkins B.L., Lipsky B.A., Hughes H.C., Bose D., Kümin M. (2019). Oral versus intravenous antibiotics for bone and joint infection. N. Engl. J. Med..

[22] Osmon D.R., Berbari E.F., Berendt A.R., Lew D., Zimmerli W., Steckelberg J.M., Rao N., Hanssen A., Wilson W.R., Infectious Diseases Society of America (2013). Diagnosis and management of prosthetic joint infection: Clinical practice guidelines by the Infectious Diseases Society of America. Clin. Infect. Dis..

[23] Berbari E.F., Kanj S.S., Kowalski T.J., Darouiche R.O., Widmer A.F., Schmitt S.K., Hendershot E.F., Holtom P.D., Huddleston P.M., Petermann G.W. (2015). 2015 Infectious Diseases Society of America (IDSA) clinical practice guidelines for the diagnosis and treatment of native vertebral osteomyelitis in adults. Clin. Infect. Dis..

[24] von Elm E., Altman D.G., Egger M., Pocock S.J., Gøtzsche P.C., Vandenbroucke J.P., STROBE Initiative (2007). The Strengthening the Reporting of Observational Studies in Epidemiology (STROBE) statement: Guidelines for reporting observational studies. Ann. Intern. Med..

[25] Diers M., Beschauner J., Felsberg M., Kossack L.I., Zeh A., Delank K.-S., Gutteck N., Werneburg F. (2025). Implementing a standard operating procedure is associated with improved vancomycin target attainment in bone and joint infections: A pre-post study. Antibiotics.

[26] Metsemakers W.-J., Morgenstern M., Senneville E., Borens O., Govaert G.A.M., Onsea J., Depypere M., Richards R.G., Trampuz A., Verhofstad M.H.J. (2020). General treatment principles for fracture-related infection: Recommendations from an international expert group. Arch. Orthop. Trauma Surg..

[27] Goldstein S.L., Kirkendall E., Nguyen H., Schaffzin J.K., Bucuvalas J., Bracke T., Seid M., Ashby M., Foertmeyer N., Brunner L. (2013). Electronic health record identification of nephrotoxin exposure and associated acute kidney injury. Pediatrics.

[28] Mehta R.L., Kellum J.A., Shah S.V., Molitoris B.A., Ronco C., Warnock D.G., Levin A., Acute Kidney Injury Network (2007). Acute Kidney Injury Network: Report of an initiative to improve outcomes in acute kidney injury. Crit. Care.

[29] Khwaja A. (2012). KDIGO clinical practice guidelines for acute kidney injury. Nephron Clin. Pract..

