# Supplementary material for: Cefazolin Versus Flucloxacillin for Methicillin-Susceptible Staphylococcus aureus Bone and Joint Infections: A Retrospective Single-Center Comparative Study of Effectiveness and Renal Safety of Intravenous Monotherapy
Source: Antibiotics (Basel). 2026 Aug 17;15(8):798. doi: 10.3390/antibiotics15080798 (PMC13509468; doi:10.3390/antibiotics15080798)
Supplement: Supplementary file 1 [file antibiotics-15-00798-s001.zip › antibiotics-4472834-supplementary.pdf]

Supplementary Materials

Cefazolin versus Flucloxacillin for Methicillin-Susceptible *Staphylococcus aureus* Bone and Joint Infections: A Retrospective Single-Center Study of the Comparative Effectiveness and Renal Safety of Intravenous Monotherapy

Felix Werneburg, Juliane Beschauner, Laura Isabell Werneburg, Alexander Zeh, Natalia Gutteck and Karl-Stefan Delank

Table S1. Sensitivity analysis of principal endpoints stratified by implant status. Odds ratios (OR; Haldane–Anscombe correction, Woolf confidence intervals) are expressed for flucloxacillin versus cefazolin; for clinical success, the odds ratio refers to clinical failure (death or not infection-free), so that values > 1 favor cefazolin. p-values from Fisher's exact test.

**Endpoint**	**Cefazolin**	**Flucloxacillin**	**OR (95% CI)**	**p**
Implant-associated infection (n = 63)	n = 40	n = 23		
30-day clinical success	38 (95.0%)	20 (87.0%)	2.63 (0.48–14.53)	0.35
1-year clinical success	33 (82.5%)	16 (69.6%)	2.03 (0.63–6.55)	0.35
Peri-treatment AKI (stage ≥ 1)	9/39 (23.1%)	5/23 (21.7%)	0.95 (0.29–3.16)	1.00
Native infection (n = 47)	n = 24	n = 23		
30-day clinical success	19 (79.2%)	17 (73.9%)	1.32 (0.36–4.86)	0.74
1-year clinical success	18 (75.0%)	14 (60.9%)	1.86 (0.56–6.26)	0.36
Peri-treatment AKI (stage ≥ 1)	3/24 (12.5%)	9/23 (39.1%)	4.02 (1.00–16.24)	0.05

Implant-associated: prosthetic joint infection and fracture-related/implant-associated infection; native: septic arthritis, native vertebral osteomyelitis and long-bone osteomyelitis. Denominators for peri-treatment AKI reflect evaluable patients. Mantel–Haenszel odds ratios pooled across strata: one-year clinical failure 2.00 (95% CI 0.84–4.75); peri-treatment AKI 1.84 (95% CI 0.75–4.49). Treatment-by-stratum interaction: p = 0.94 (one-year clinical failure) and p = 0.11 (AKI). All analyses were exploratory. AKI, acute kidney injury; CI, confidence interval; OR, odds ratio.
